# Recurrent sick leave after COVID-19: investigating the first wave of the pandemic in a comprehensive Swedish registry-based study

**DOI:** 10.1186/s12889-021-11918-y

**Published:** 2021-10-21

**Authors:** Annie Palstam, Emma Westerlind, Katharina S. Sunnerhagen, Hanna C. Persson

**Affiliations:** Department of Clinical Neuroscience, Institute of Neuroscience and Physiology, Sahlgrenska Academy, University of Gothenburg, and Sahlgrenska University Hospital, Per Dubbsgatan 14, 3 tr, 413 46 Gothenburg, Sweden

**Keywords:** Sickness benefits, Return to work, Post-COVID, COVID-19, Registries

## Abstract

**Background:**

Sick-leave due to COVID-19 vary in length and might lead to re-current episodes. The aim was to investigate recurrent sick leave due to COVID-19 during the first wave.

**Methods:**

This is a registry-based cohort study. The study comprises all people with sickness benefit due to COVID-19 in Sweden in March 1–August 31, 2020. Data from the Swedish Social Insurance Agency, the Swedish National Board of Health and Welfare, and Statistics Sweden were merged.

**Results:**

Within the follow-up period of 4 months, 11,955 people were subject to sickness benefit due to COVID-19, whereof 242 people (2.0%) took recurrent sick leave due to COVID-19, and of those 136 (56.2%) remained on sick leave at the end of follow-up. People with recurrent sick leave were older, more often women, and more likely to have been on sick leave prior to the COVID-19 pandemic.

**Conclusion:**

A group of people presented with recurrent sick leave due to COVID-19. For half of them, the second sick leave lasted throughout the follow-up. People with recurrent sick leave differ in several aspects from those with shorter sick leave. To capture long-term sick-leave patterns due to COVID-19, a longer period of follow-up is needed.

**Supplementary Information:**

The online version contains supplementary material available at 10.1186/s12889-021-11918-y.

## Introduction

All of society is affected by the COVID-19 pandemic. The long-term consequences are immeasurable for affected persons and their families, as well as for the economy, public health, health care, and the health insurance system. Furthermore, the symptoms of COVID-19 are diverse, including fever, cough, myalgia, and fatigue etc., and the severity, and functional impairments vary over time [1–6]. Post infection, rehabilitative actions to improve the ability of people to return to ordinary routines, including work, are needed.

Sick leave is an indicator of well-being in the working-age population. In Sweden, sick-leave rates almost doubled during March and April 2020 compared with the previous year [7]. The financial compensation for sick leave is tax-funded and comprehensive in Sweden. However, the availability of paid sick leave varies globally [6]. National diagnosis-specific guidelines on sick leave are provided by the Swedish Social Insurance Agency and the National Board of Health and Welfare, and since June 2020 they have included COVID-19. Due to an insufficient knowledge base, the guidelines for COVID-19 are vague, but they do acknowledge that an affected person’s ability to work could decline not only in the acute phase but also in the aftermath [8].

Symptoms related to COVID-19, such as fatigue, dyspnoea, pain and depression, can be protracted and require intensive use of medical resources, regardless of the severity of the illness in the acute phase [9–13]. In our recent study on sick leave due to COVID-19 during the first wave in Sweden, 9% were on sick leave at the end of the 4-month follow-up period [14]. However, recurrent sick leave was not investigated, and studies on recurrent sick leave due to COVID-19 has to our knowledge not been presented elsewhere.. The aim of this study was to investigate recurrent sick leave in people initially returning to work during the first wave of COVID-19, in terms of days, and differences compared to those with one shorter period of sick leave, in a national comprehensive cohort.

## Methods

This is a registry-based study with data from the Swedish Social Insurance Agency, the Swedish National Board of Health and Welfare, and Statistics Sweden, based on the unique Swedish personal identification number. The personal identification numbers were used to pool data from different registries in the present study, and researchers had access only to a pseudonymised data set. A patient has been involved in the study as a partner in research, contributing to the research question and discussion of the results.

### Study population

All people who were registered as receiving sickness benefits due to a COVID-19 diagnosis (International Statistical Classification of Diseases ICD [15] code U07) in Sweden from March 1 through August 31, 2020, were included in this study. Codes U (U00-U49) are used by WHO for provisional assignment of new diseases of uncertain etiology (https://icd.who.int/browse10/2019/en#/U07.1).

The follow-up period was 4 months (122 days) from the start of sick leave. Sickness benefits can be granted from the Swedish Social Insurance Agency to everyone who has been working in Sweden (both employed and self-employed) and also during parental leave, studies, and registered unemployment. If the person is employed, the employer pays sick pay for the first 2 weeks of absence due to sickness, and thereafter sickness benefits are provided. In the present study, sick leave is defined as receiving sickness benefits, regardless of amount.

### Sick leave

The sick-leave period due to COVID-19 was counted as the number of days with sickness benefits and had to include at least one registration with a COVID-19 diagnosis. If sick pay was received in the first 2 weeks, these weeks were included in the sick leave period. If two sick leave periods were separated by a gap of ≤14 days, they were merged into one period. Registered diagnoses related to COVID-19 that were merged into the sick leave period if they were registered within 14 days from the start or end of the COVID-19 sick leave are presented in Table 1.

**Table 1:** Diagnoses related to COVID-19

Diagnoses related to COVID-19	ICD codes
Acute bronchitis	J20.9
Acute lower respiratory infection	J22, J22.9
Acute laryngopharyngitis/acute upper respiratory infection	J06, J06.8, J06.9
Acute laryngitis	J04.0
Asthma	J45, J45.9
Acute sinusitis	J01.9
Influenza due to unidentified influenza virus	J11, J11.8
Chronic tonsillitis	J35.0
Viral infection	B34.2, B34.8, B34.9, B34-P
Sequelae of infectious and parasitic disease*	B94
Postviral fatigue syndrome*	G93.3
Fever	R50.9
Malaise and fatigue	R53, R53.9
Headache	R51.9
Haemoptysis	R04.2
Deep vein thrombosis*	Z86.7B
Family history of asthma and other chronic lower respiratory disease	Z82.5
Nonrheumatic aortic (valve) insufficiency*	I35.1
Anxiety disorder*	F41.0, F41.9, F41.9P
Reaction to stress*	F43.0, F43.8, F43.8A
Depressive disorder*	F32.1, F32.9

*Abbreviation*: *ICD* International Statistical Classification of Diseases

*  =  related to COVID-19 only if it was registered after the COVID-19 diagnosis (U07)

Sick leave prior to COVID-19 was defined as being registered with sick leave for ≥28 days or ≥ 6 times between March 1, 2019, and the start of COVID-19 sick leave.

### Recurrent sick leave due to COVID-19

Recurrent sick leave was defined as re-entry into sick leave due to COVID-19 (U07) or postviral fatigue syndrome (G93.3) with a gap of > 14 days from the end of the first sick leave period, within the follow-up period.

The group with recurrent sick leave was compared with the sub-group in the total population that had short sick leave, which was defined as a period shorter than the median number of sick leave days for the total population (i.e., < 35 days).

### Variables

Data on employment status were retrieved from the Swedish Social Insurance Agency. The subgroups were employment (including parental leave, and combined employment and self-employment), self-employment, and unemployment (including studies).

Socioeconomic variables were collected from Statistics Sweden. Educational level was presented as primary school (≤9 years), secondary school (10–12 years), short university education (13–14 years), and long university education (≥15 years). Country of birth was categorised into Sweden, European countries except for Sweden, and countries outside of Europe. Marital status categories were married (including both marriage and registered partnership), single, and divorced and widow/widower. Income was reported in thousands of Swedish krona (SEK), 1 Euro = 10.18 SEK on March 25, 2021, and was the disposable income for each person.

Data on in-hospital care due to COVID-19 were retrieved from the Swedish National Board of Health and Welfare. In-hospital care due to COVID-19 was defined as at least 1 day of hospital stay with any of the U07 (COVID-19) diagnoses being registered. In cases where the COVID-19 diagnosis was a secondary diagnosis, the primary diagnosis is presented in Supplementary Table 1. The National Board of Health and Welfare furthermore provided information about the date of death during the study period, when applicable.

### Statistical methods

**Fig. 1:**
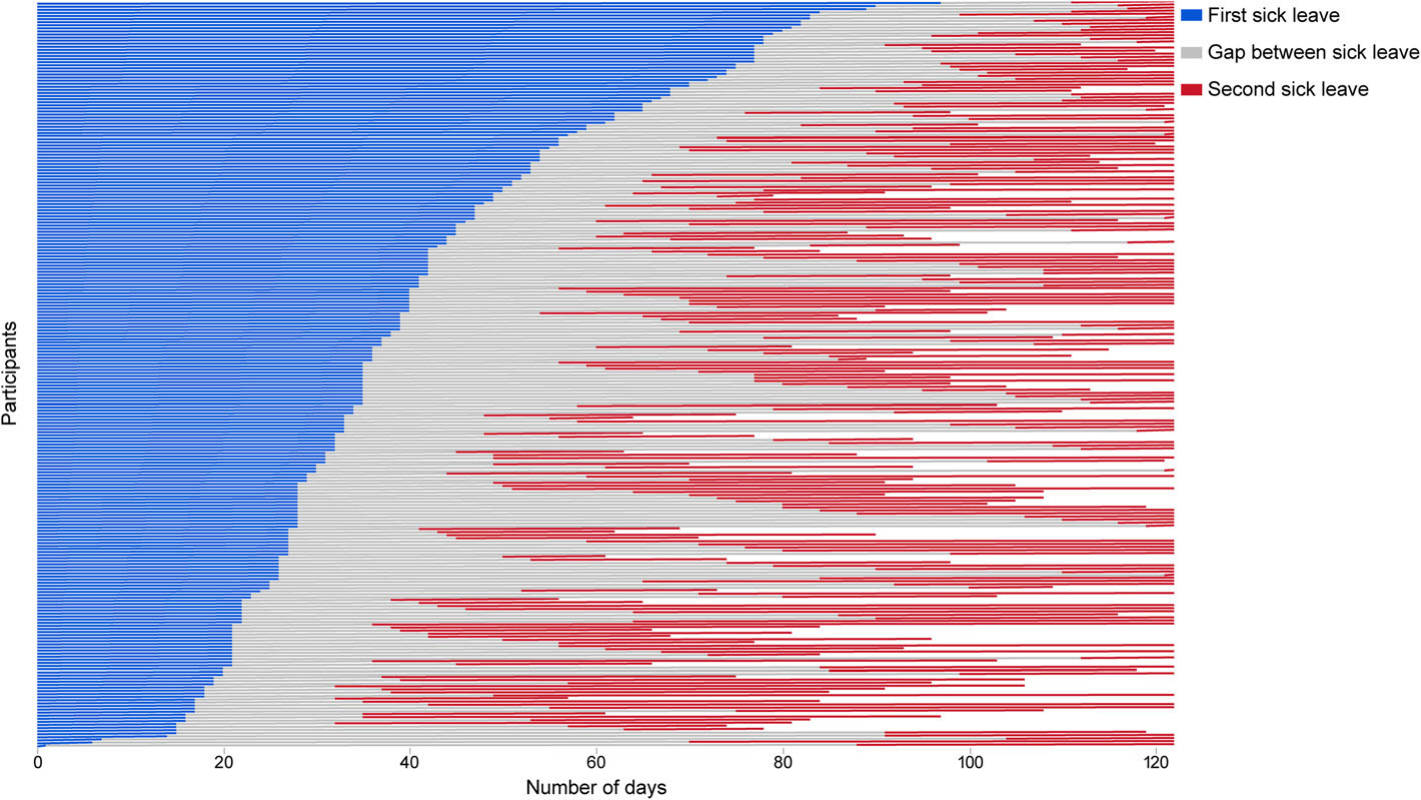
Recurrent sick leave in 242 people during the four month follow-up period. The white areas indicate no longer being on sick leave

SPSS Statistics 25 (IBM) was used to manage and analyse the data. Fisher’s exact test and the Mann-Whitney U test were used to analyse differences between groups in categorical and continuous variables, respectively. The significance level was set to *p* < 0.05. To graphically present periods of sick leave in the group experiencing recurrent sick leave, a lasagne plot (Fig. 1) was created in SAS JMP 15. In cases of death during the study period, the person was registered as being on sick leave for the total time of the follow-up period.

## Results

A total of 11,955 people were granted sickness benefits due to COVID-19 from March 1 through August 31, 2020, in Sweden. Characteristics of the study population are presented in Table 2. The median number of days on sick leave was 35 (IQR 26). For a sub-group of 1073 people (9.0%), the sick leave continued throughout the 4 month follow-up period. Twenty-four people (0.2%) died before the end of follow-up, all within < 10 days from the end of the sick leave period due to COVID-19.

**Table 2:** Characteristics of the study population

Participants, n (%)	11,955 (100.0)
Sex, n (%)	
Men	4826 (40.4)
Women	7129 (59.6)
Age, mean (SD)	48.0 (11.3)
Country of birth, n (%)^a^	
Sweden	7545 (63.1)
European countries except for Sweden	1481 (12.4)
Countries outside of Europe	2929 (24.5)
Educational level, n (%)^b^	
Primary school (≤9 years)	1237 (10.4)
Secondary school (10–12 years)	5889 (49.6)
Short university education (13–14 years)	1743 (14.7)
Long university education (≥15 years)	3995 (25.2)
Income, 1000 SEK median (IQR)^c^	288 (117)
Marital status, n (%)^d^	
Married	5812 (48.8)
Single	3859 (32.4)
Divorced and widow/widower	2246 (18.8)
Sick leave prior to COVID-19, n (%)	1932 (16.2)
Employment status, n (%)^e^	
Employment	11,460 (95.9)
Self-employment	288 (2.4)
Unemployment	204 (1.7)
In-hospital care due to COVID-19, n (%)	2960 (24.8)

*Abbreviation*: *SD* standard deviation, IQR interquartile range

a  =  9 missing, b  =  91 missing, c  =  2 missing, d  =  38 missing, e  =  3 missing

Two hundred and forty-two people (2.0%) had recurrent sick leave. For the second sick leave period, 133 (55.0%) were registered as U07.1 (COVID-19, virus identified), 76 (31.4%) as U07.2 (COVID-19, virus unidentified), and 33 (13.6%) as G93.3 (postviral fatigue syndrome). In addition, three people had a third sick-leave period due to COVID-19 within the follow-up period.

The median number of days of the first sick leave period for the group with recurrent sick leave was similar to that of the total population, which was 35 (IQR 24) (Fig. 1). The median number of days between the initial and the recurrent sick-leave periods due to COVID-19 was 33.5 (IQR 31). The median number of days of the second sick-leave period was 26 (IQR 26) at the end of follow-up. A total of 136 (56.2%) people with recurrent sick leave were still on sick leave at the end of the 4-month follow-up.

People with recurrent COVID-19 sick leave were significantly older and more often women than people with one shorter period of sick leave (< 35 days) (Table 3). Furthermore, the group with recurrent sick leave was more likely to have been on sick leave prior to COVID-19, and there were significant differences in country of birth, educational level, employment status, marital status, and income. There was no significant difference in in-hospital care due to COVID-19 between the two groups (Table 3).

**Table 3:** Characteristics of the work instability group compared with the group of people with short sick leave (< 35 days) due to COVID-19

	Group with recurrent sick leave	Group with one shorter period of sick leave	*p*-value
Participants, n	242	5343	
Sex, n (%)			0.011
Men	77 (31.8)	2143 (40.1)	
Women	165 (68.2)	3200 (59.9)	
Age in years, mean (SD)	48.7 (10.4)	46.9 (11.4)	0.023
Country of birth, n (%)			0.015
Sweden	177 (73.1)	3418 (64.0)	
European countries except for Sweden	23 (9.5)	650 (12.2)	
Countries outside of Europe	42 (17.4)	1270 (23.8)	
Educational level, n (%)			0.001
Primary school (≤9 years)	25 (10.3)	507 (9.6)	
Secondary school (10–12 years)	99 (40.9)	2672 (50.5)	
Short university education (13–14 years)	28 (11.6)	772 (14.6)	
Long university education (≥15 years)	90 (37.2)	1342 (25.4)	
Income: 1000 SEK median (IQR)	276 (103)	289 (117)	0.042
Marital status, n (%)			0.006
Married	95 (39.3)	2576 (48.3)	
Single	88 (36.4)	1831 (34.3)	
Divorced and widow/widower	59 (24.4)	929 (17.4)	
Sick leave prior to COVID-19, n (%)	55 (22.7)	735 (13.8)	< 0.001
Employment status, n (%)			0.030
Employment	225 (93.4)	5160 (96.6)	
Self-employment	9 (3.7)	105 (2.0)	
Unemployment	7 (2.9)	77 (1.4)	
In-hospital care due to COVID-19, n (%)	46 (19.0)	966 (18.1)	0.733

## Discussion

In this national registry-based study, comprising all people on sick leave during the first wave of COVID-19 in Sweden, 2% had recurrent sick leave during the 4-month follow-up period. The second period of sick leave varied in length and point in time within the follow-up period, and over half of the population with recurrent sick leave was still on sick leave at the end of follow-up, indicating a need for a longer period of follow-up to explore long-term sick leave patterns. The group of people with recurrent sick leave due to COVID-19 differed in several aspects from the group of people with one shorter sick leave.

There were 136 people with recurrent sick leave who were still on sick leave at the end of the 4-month follow-up period. Increasing sick-leave rates have been observed during influenza epidemics, a recent example being the H1N1 epidemic [16]. However, reports of previous pandemics of viral infections resulting in long-term sick leave or recurrent sick leave are lacking. In our previous study [14] on the same cohort (*n* = 11,955), we showed that 9% (*n* = 1073) of the population were on their initial sick leave for the entire follow-up period. Taking into account recurrent sick leave, the number of people with longer periods of sick leave due to post COVID-19 symptoms is probably higher than previously reported. It can also be speculated that a longer follow-up period could reveal more people registered with recurrent sick leave at a later phase. It seems that long-term follow-up is needed to attain the full picture of the effect COVID-19 has had on public health.

People with recurrent sick leave due to COVID-19 were more likely to be older, female, and to have had prior sick leave during the prior year than those who had one shorter period of sick leave. However, the groups were similar regarding in-hospital care due to COVID-19. The patterns of sick leave may depend on several circumstances. The Swedish national guidelines for sick leave due to COVID-19 were first published in June 2020 and are still under development [17], and this could influence the country’s sick leave patterns. Furthermore, the government regulations on quarantine due to COVID-19 and available testing have varied since the pandemic began. Recurrent periods of sick leave could be due to persistent symptoms or onset of new symptoms post COVID-19 [18]. Recurrent sick leave could also be due to return to work with reduced work capacity, which has been reported as a risk for future sick leave in other diseases but not yet in COVID-19 [19]. Occupational characteristics, such as a low level of adjustment latitude, could also hinder return to work [20].

This study included only people with sickness benefits. In Sweden, this generally includes people with a sick-leave period of more than 2 weeks. Furthermore, the cohort included few deaths compared with national age-matched data [21], which may be explained by the risk of people dying prior to being registered with sickness benefits for COVID-19.

An important methodological consideration is the definition of COVID-19 sick-leave periods. Approximately one-third of the people were classified as “COVID-19, virus undetected”, indicating uncertainty in the data. Data were collected during the first wave in Sweden, when PCR testing was limited. In the present study, the “COVID-19, virus undetected” diagnosis was classified as a COVID-19 infection, to capture the picture of sick leave due to COVID-19. Sick leave due to related diagnoses were merged into the period of COVID-19 sick leave if the gap between sick leaves was shorter than 14 days. The 14-day cut-off was based on clinical experience and reasoning, to limit the risk of missing substantial sick leave data due to registration difficulties (first-wave data, early in the pandemic). However, there may be a risk that a small number of the sick-leave periods were due not only to COVID-19. The cut-off also affects the results, in that the shortest time possible between the initial and the recurrent sick leave was 14 days.

It is clear that a period of 4 months is too short to give a full and comprehensive view of the sick-leave patterns over time in this complex and heterogeneous disease, and longer periods of follow-up are needed in order to capture data on sustainable return to work in this population. Furthermore, sick leave and the ability to work are closely related to an individual’s occupation and type of work, which are unknown characteristics in this cohort.

To our knowledge this is the first national registry–based cohort study describing recurrent sick leave due to COVID-19. Information on sick-leave patterns from the present study could be useful for guidelines and recommendations regarding sick leave and rehabilitation needs due to COVID-19.

## Conclusion

In this national cohort with people receiving sickness benefits in the first wave of the COVID-19 pandemic in Sweden, a group of people presented with recurrent sick leave due to COVID-19. The group with recurrent sick leave differs in several aspects from the group with shorter sick leave. For half of them, the second sick leave lasted throughout the follow-up period. People with sick leave prior to COVID-19 seem to be vulnerable to recurrent sick leave, and may need early attention and support in vocational rehabilitation. A longer follow-up period is needed to capture long-term sick-leave patterns due to COVID-19.

## Supplementary Information

**Additional file 1: Table S1.** Primary diagnosis for inpatient care if not COVID-19.

## Data Availability

The datasets used and/or analysed during the current study are available from the corresponding author on reasonable request. They are not publicly available, in accordance with the Ethics Review Authority. The study protocol and statistical analysis plan are available at https://www.researchweb.org/is/vgr/project/274476.

## Abbreviations

SD: Standard deviations
IQR: Interquartile range
