# Supplementary material for: Recurrent sick leave after COVID-19: investigating the first wave of the pandemic in a comprehensive Swedish registry-based study
Source: BMC Public Health. 2021 Oct 21;21:1914. doi: 10.1186/s12889-021-11918-y (PMC8530010; doi:10.1186/s12889-021-11918-y)
Supplement: Supplementary file 1 — Additional file 1: Table S1. Primary diagnosis for inpatient care if not COVID-19. [file 12889_2021_11918_MOESM1_ESM.docx]

Supplemental Table

Table SI. Primary diagnosis for inpatient care if not COVID-19

| Primary diagnosis if not COVID-19 | ICD codes |
| --- | --- |
| Cardiovascular diseases | I25, I26, I30, I48 |
| Respiratory disorders | J03, J12, J16, J17, J18, J80, J99 |
| Diseases of the abdominal organs | A09, K35, K45, K57, K80, K81, K85, K92 |
| Kidney disease | N10, N19 |
| Brain disorders | I63, G40, G93 |
| Complications during pregnancy, childbirth, and puerperium | O00, O26, O44, O47, O80, O98 |
| Viral infection | B34 |
| Symptom diagnoses | R00, R05, R06, R10, R20, R41, R50, R55 |
| Other diagnoses | T81, S82, R73, R94, D50, L03, E10, E11, F41 |

Abbreviation: ICD: International Classification of Diseases.
